# Adaptations in Muscle Activity to Induced, Short-Term Hindlimb Lameness in Trotting Dogs

**DOI:** 10.1371/journal.pone.0080987

**Published:** 2013-11-13

**Authors:** Stefanie Fischer, Ingo Nolte, Nadja Schilling

**Affiliations:** 1 University of Veterinary Medicine Hannover, Foundation, Small Animal Clinic, Hannover, Germany; 2 Friedrich-Schiller-University, Institute of Systematic Zoology and Evolutionary Biology, Jena, Germany; University of Sydney, Australia

## Abstract

Muscle tissue has a great intrinsic adaptability to changing functional demands. Triggering more gradual responses such as tissue growth, the immediate responses to altered loading conditions involve changes in the activity. Because the reduction in a limb’s function is associated with marked deviations in the gait pattern, understanding the muscular responses in laming animals will provide further insight into their compensatory mechanisms as well as help to improve treatment options to prevent musculoskeletal sequelae in chronic patients. Therefore, this study evaluated the changes in muscle activity in adaptation to a moderate, short-term, weight-bearing hindlimb lameness in two leg and one back muscle using surface electromyography (SEMG). In eight sound adult dogs that trotted on an instrumented treadmill, bilateral, bipolar recordings of the m. triceps brachii, the m. vastus lateralis and the m. longissimus dorsi were obtained before and after lameness was induced. Consistent with the unchanged vertical forces as well as temporal parameters, neither the timing nor the level of activity changed significantly in the m. triceps brachii. In the ipsilateral m. vastus lateralis, peak activity and integrated SEMG area were decreased, while they were significantly increased in the contralateral hindlimb. In both sides, the duration of the muscle activity was significantly longer due to a delayed offset. These observations are in accordance with previously described kinetic and kinematic changes as well as changes in muscle mass. Adaptations in the activity of the m. longissimus dorsi concerned primarily the unilateral activity and are discussed regarding known alterations in trunk and limb motions.

## Introduction

Muscle is one of the most plastic tissues in the animal body. Its great phenotypic plasticity allows it to adapt to various tasks and respond to changing functional demands throughout life (reviewed in [1]). Immediate responses to altered functional requirements involve, for example, changes in muscle recruitment and activation patterns, while more gradual adaptations include quantitative and qualitative changes in gene expression as well as tissue growth and remodeling [2,3]. When diseased or injured, however, an animal must immediately respond and this is first and foremost accomplished by adaptations in muscle recruitment. 

Animals have evolved compensatory strategies to cope with the partial loss of limb function. The associated lameness is marked by deviations of the animal’s gait from the physiological pattern. Locomotor adaptations to lameness include changes in kinetics and kinematics as well as muscle activity. The changes in the ground reaction forces (GRF) or the motion patterns are comparatively well established (e.g., hindlimb lameness in dogs [4–17]:, respectively), whereas adaptations in muscle activity have only been marginally studied [18]. Nonetheless, the observed redistribution of body weight and the dynamic shift of the position of the center of body mass (CoM) alter the loading of the limbs and the trunk, which must be met and are accomplished by changes in muscle function. 

To gain insight into the changes in muscles function in adaptation to reduced limb loading, we recorded the activity of two limb and one back muscle in dogs before and after a moderate weight-bearing hindlimb lameness was induced. Bipolar surface electromyography (SEMG) recordings were obtained bilaterally while the dogs trotted on a horizontal treadmill. To accommodate changes in the work performed and the forces transmitted, muscle recruitment may be modulated in its timing and/or intensity [19]. Therefore, we evaluated the following parameters: 1) onset and offset of the activity as well as the timing of peak activity and 2) maximum activity and integrated SEMG area. The two limb muscles examined -m. triceps brachii, m. vastus lateralis- are part of the extensor group of the elbow and knee that serve to resist gravity (i.e., ‘antigravity muscles’; [20]). Because changes in limb loading should be reflected by changes in these muscles’ activities, we expected the greater vertical force reported for the hindlimb contralateral to the affected limb ([9] and references therein) to result in an increased activity of the contralateral m. vastus lateralis. Conversely, the reduced loading of the affected hindlimb should be associated with a decreased activity of the ipsilateral m. vastus lateralis. Because the results from our related study indicated no significant change in the weight-bearing characteristics of the forelimbs [9], we hypothesized that the activity of the m. triceps brachii would not significantly change. 

As in other quadrupeds, the epaxial muscles of dogs play a central role in stabilizing and mobilizing the trunk; that is, they stabilize the trunk against inertial loading, provide a foundation for the production of mechanical work by the limbs, and integrate the coordinated action of the limbs [21-23]. Particularly the lumbar muscles function to provide a firm base for extrinsic hindlimb muscle action by stabilizing the pelvis and controlling the forces transmitted between the limbs and the trunk [24]. Because in animals showing lameness, both the forces exerted by the limbs as well as pelvic and truncal motions are altered, we expected the activity of the lumbar epaxial muscles (i.e., the m. longissimus dorsi) to be significantly different after lameness was induced. 

## Materials and Methods

### Ethics statement

Data collection for this study was carried out in strict accordance with the German animal welfare guidelines. All experiments were approved by the ethics committee of the State of Lower Saxony (No 12/0717). 

### Animals and experimental design

Eight adult and clinically sound individuals (7 males, 1 female; mean±SD: 4±1 years; 15.1±1.2 kg) of the Beagle population of the Small Animal Clinic of the University of Veterinary Medicine Hannover Foundation (Germany) were enrolled in this study. The simultaneously recorded ground reaction forces as well as the previous clinical examination confirmed that the dogs were sound [9].

After habituation, data collection started as soon as the dogs trotted smoothly and comfortably on the horizontal four-belt treadmill equipped with a force plate underneath each belt (Model 4060-08, Bertec Corporation). Based on our previous studies [9,25,26], treadmill speed was set at 1.4 m/s for both lame and sound trials. This speed represented the preferred trotting speed of the dogs, which was confirmed during their habituation period. At their preferred speed, the dogs trotted most comfortably and matched the treadmill speed with ease and without accelerating or decelerating [27,28]. Control data were collected after warm-up and before a reversible moderate supporting lameness was induced in the right hindlimb by evoking pressure on the paw sole (reduction in peak vertical force: 33±9%). After collecting the control data and a break of approximately 15 min, lameness was induced using a small sphere of 9.5 or 16 mm in diameter, which was coated with cotton and taped under the paw (for details, see 26). Both control and lameness data comprised at least 5-10 trials, each lasting up to 30 s and covering between 48 and 65 strides. The body side on which lameness was induced is hereafter referred to as ipsilateral in contrast to the contralateral, sound body side. After data collection, the dogs ambulated on the treadmill again without any sign of residual lameness. 

### Data collection

Bipolar recordings were obtained bilaterally from the m. triceps brachii, the m. vastus lateralis and the m. longissimus dorsi using SEMG. After gentle preparation of the skin (i.e., clipping, shaving, cleaning, degreasing), disposable Ag-AgCl electrodes with a circular uptake area of 1.6 cm in diameter and an interelectrode distance of 2.5 cm were applied (H93SG, Arbo). Electrode placement was the same for each individual before and after lameness was induced because the data for the control and the lame conditions were recorded in the same session. The same experimenter (SF) applied all electrodes to ensure consistency in the recording sites between body sides and among individuals. 

**Figure 1:**
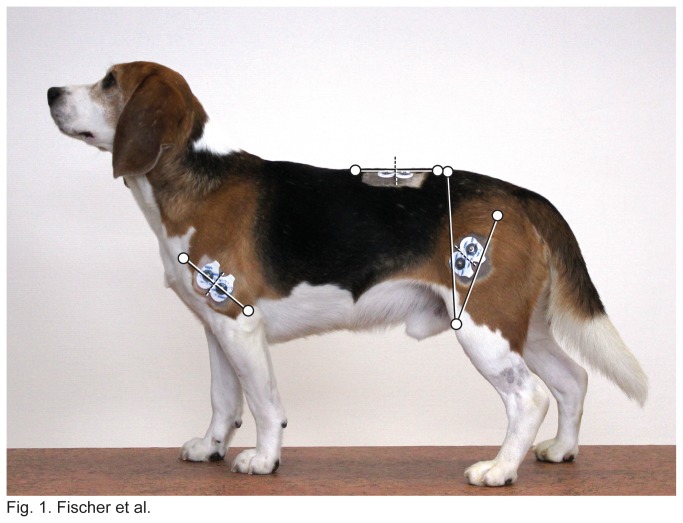
One of the subjects partially instrumented to illustrate the electrode positioning (for details on the skeletal landmarks, see Material & Methods).

For the m. vastus lateralis, electrode placement followed the recommendations by Bockstahler and colleagues [29]. That is, the midpoints of the lines connecting the posterior superior iliac spine and the patella and the patella and the trochanter major were determined. Then, the electrodes were placed dorsal and ventral of the line connecting these two midpoints (Figure 1). Electrodes for the m. triceps brachii were positioned halfway along the line connecting the Tuberculum *majus* humeri and the olecranon. Muscle activity of the m. longissimus dorsi was recorded at the lumbar level L3/L4. Electrode location was midway between the vertebral articulation of the last rib and the most cranial aspect of the iliac crest and about a finger’s breadth lateral from the spinous processes. After placement, the electrodes were connected to the transmitters that transferred the signal to a PC (Zero wire EMG, Aurion). Electromyographic signals were recorded simultaneously with the GRF using Vicon Nexus (Vicon motion systems Ltd.). Data were sampled at 2,000 Hz, amplified 1,000 times and collected in the range from 10 Hz to 1.000 Hz. The transmitters were carefully secured with tape and hair clips to minimize motion artifacts. Nevertheless, not all recordings could be evaluated in each individual. 

### Data analysis

To allow for the direct comparison between muscle activity and limb function, indicated by its weight-bearing characteristic, the same 10 strides as evaluated in a previous study were analyzed herein [9]. Touch down and lift off were manually defined in Vicon Nexus using the vertical component of the GRF (sampling rate 1,000 Hz); force threshold was set at 13 N. SEMG data were high-pass filtered at 20 Hz, low-pass filtered at 300 Hz, and subsequently smoothed using a moving average with a sample window of 10 ms. The sampled data per stance and swing phase varied slightly in duration during a trial and therefore differed in the number of recorded data points. To enable comparisons of the activity with reference to footfall events, the SEMG signals were time-normalized to the same number of data points (i.e., bins) per stance and swing phase (i.e., each phase covered 50% of the stride cycle resulting in altogether 201 bins per sampled stride; for details see [19]). These filtered and time-normalized data were then exported to Microsoft Excel for further analysis. 

To evaluate differences in timing and intensity of the muscle activity, data analysis follows previously established protocols (see [19] and references therein). Briefly, SEMG signals were amplitude-normalized using the muscle’s average activity during the sound condition. For this, the mean activity was determined for the control data and then, each bin of both the sound and the lame trials was divided by this mean. By normalizing the values for each dog to the mean activity of the control prior to generating the statistics for all dogs, the pattern from one dog did not overwhelm the pattern from another (e.g., because of differences in signal strengths due to different skin properties etc.). From these data, grand averaged curves were calculated per muscle and dog.

For each muscle and dog, the timing of the peak activity was compared between conditions. Furthermore, on- and offset times of the muscle activity were determined using a threshold that was twice the baseline activity of the control data. For this, first, baseline activity was established by averaging the values from a fraction (i.e., 20 bins) of the stride cycle when the muscle was inactive. This period of inactivity was determined by visual inspection and covered the values between 75% and 84% of the stride cycle in the m. triceps brachii, 39% to 49% in m. vastus lateralis and 17% to 27% (N=3) or 50% to 59% (N=2) in the m. longissimus dorsi. The respective muscle was considered active when the SEMG amplitude was above this threshold for 7.5±9.5% of the stride cycle (i.e., 15±20 bins); except the ipsilateral m. vastus lateralis, for which a longer period was chosen because of the greater fluctuations of the EMG signal. Conversely, muscle activity ended when the amplitude was below the threshold for 7.0±5.0% of the stride cycle (i.e., 14±10 bins). The period of time the activity had to be above or below the threshold varied somewhat among subjects and muscles due to differences in the baseline activity and was therefore crosschecked visually. Note that because of the very low level and the stark fluctuations of the swing phase activity of the m. vastus lateralis, this activity could not be analyzed quantitatively. It was therefore excluded from the statistical comparisons. 

Additionally to comparing the timing of the muscle activity, recruitment intensity was compared between control and induced-lameness data using peak activity and integrated SEMG area (i.e., the sum of the values of the bins in the phase- and amplitude-normalized signal when the muscle was active). To further specify significant differences in muscle activity between sound and lame conditions, we compared the signal on a bin-by-bin basis. For this, the difference between the muscle’s activity in the sound and the lame condition was calculated and then compared with the hypothesized difference of zero by computing 97.5th and 2.5th percentiles of the difference when averaged across dogs. If these percentiles encompassed zero, the null hypothesis was accepted. If they failed to encompass zero (i.e., both 97.5th and 2.5th percentiles were greater than or less than zero), the null hypothesis was rejected and the change in muscle activity across conditions significant. 

### Statistical analyses

For all EMG values, mean±standard deviation are presented in the following (see also Table 1). Wilcoxon signed rank tests were used to compare integrated SEMG area, peak activity as well as the timing of the muscle activity. Because of the lower sample size, paired t-tests were used to compare the data for the m. longissimus dorsi. P values of p<0.05 indicate significant differences, but note that due to the lower sample size (e.g., ascribable to motion artifacts), these differences need to be interpreted with caution in the m. longissimus dorsi. All statistical tests were performed in GraphPad Prism (GraphPad Software Inc.).

**Table 1:** EMG results for the muscles on the body side on which hindlimb lameness was induced (i.e., ipsilateral) and the body side opposite to the one on which hindlimb lameness was induced (i.e., contralateral).

**Ipsilateral body side**							
		**sound**	**lame**		**p**	**r**	
**M. triceps brachii**							
on		94.0±1.8	93.5±2.6		0.313	0.936	n.s.
off		71.5±3.1	71.7±3.0		0.844	0.908	n.s.
tmax		7.2±2.6	15.8±10.4		0.078	0.252	n.s.
max		2.4±0.7	2.3±0.8		0.688	0.714	n.s.
area		183.0±4.6	187.5±32.7		0.813	-0.393	n.s.
***M. vastus lateralis***							
on		85.6±2.5	83.9±2.8		0.156	0.150	n.s.
of		34.0±2.6	38.3±2.3		0.016	0.821	*
tmax		17.8±4.9	20.5±8.5		0.469	0.393	n.s.
max		2.4±0.7	1.3±0.4		0.016	0.714	*
area		108.5±18.7	92.3±26.7		0.031	0.901	*
**M. longissimus dorsi**							
on^1st^	27.9±2.0		25.8±1.9		0.160	0.063	n.s.
off^1st^	45.1±2.7		44.3±1.9		0.664	-0.288	n.s.
tmax^1st^	33.9±2.5		32.4±4.0		0.504	0.096	n.s.
max^1st^	2.9±0.4		2.7±0.5		0.083	0.870	n.s.
area^1st^	69.6±15.5		75.8±13.1		0.846	0.275	n.s.
on^2nd^	80.9±2.7		81.6±3.9		0.560	0.780	n.s.
off^2nd^	92.9±1.6		92.7±1.7		0.900	-0.907	n.s.
tmax^2nd^	85.6±2.4		86.4±2.7		0.438	0.672	n.s.
max^2nd^	1.9±0.4		1.7±0.3		0.520	0.328	n.s.
area^2nd^	36.5±11.6		32.5±7.6		0.846	0.275	n.s.
area^1+2^	106.0±20.5		108.3±19.2		0.458	0.311	n.s.
**Contralateral body side**							
	**sound**		**lame**		**p**	**r**	
**M. triceps brachii**							
on	94.6±1.9		95.0±1.6		0.313	0.782	n.s.
off	68.0±4.7		68.6±3.8		0.563	0.873	n.s.
tmax	11.9±8.7		10.3±8.4		1.000	0.391	n.s.
max	2.4±0.8		2.5±1.0		0.938	0.286	n.s.
area	183.2±4.8		209.4±31.3		0.078	0.643	n.s.
***M. vastus lateralis***							
on	85.0±2.5		88.1±1.3		0.063	-0.374	n.s.
of	31.8±5.6		40.4±6.3		0.016	0.664	*
tmax	18.1±3.0		21.7±3.9		0.016	0.546	*
max	2.2±0.3		3.0±0.8		0.031	0.643	*
area	102.1±21.4		164.0±25.2		0.016	0.198	*
**M. longissimus dorsi**							
on^1st^	26.9±1.9		29.5±3.5		0.029	0.953	*
off^1st^	45.3±1.7		46.5±2.1		0.208	0.567	n.s.
tmax^1st^	32.9±2.6		37.7±2.0		0.013	0.434	*
max^1st^	2.7±0.4		3.3±0.9		0.257	0.037	n.s.
area^1st^	70.1±6.6		74.7±12.3		0.774	0.703	n.s.
on^2nd^	81.6±2.9		83.6±3.7		0.075	0.868	n.s.
off^2nd^	93.1±2.5		94.0±2.3		0.588	-0.022	n.s.
tmax^2nd^	88.6±2.4		90.2±1.0		0.169	0.466	n.s.
max^2nd^	2.0±0.2		1.7±0.3		0.001	0.974	*
area^2nd^	36.8±7.9		29.3±15.7		0.774	0.703	n.s.
area^1+2^	106.9±12.3		104.1±26.9		0.434	0.323	n.s.

On- and offset (on, off) and timing of the peak (tmax) of the muscle activity (mean±SD in % of the stride cycle) as well as magnitude of the peak activity (max) and the integrated SEMG area (mean±SD). Note that in case of the limb muscles, only the main activity associated with the stance phase was evaluated. For the back muscle, the two bursts were analyzed separately plus the summed activity (area 1+2). Sample size was N=7 for the leg and N=5 for the epaxial muscles. Significant differences between sound and lame conditions at * p<0.05; n.s.=not significant, r=effect size (i.e., Pearson’s correlation coefficient).

## Results

### M. *triceps brachii (N=7)*

**Figure 2:**
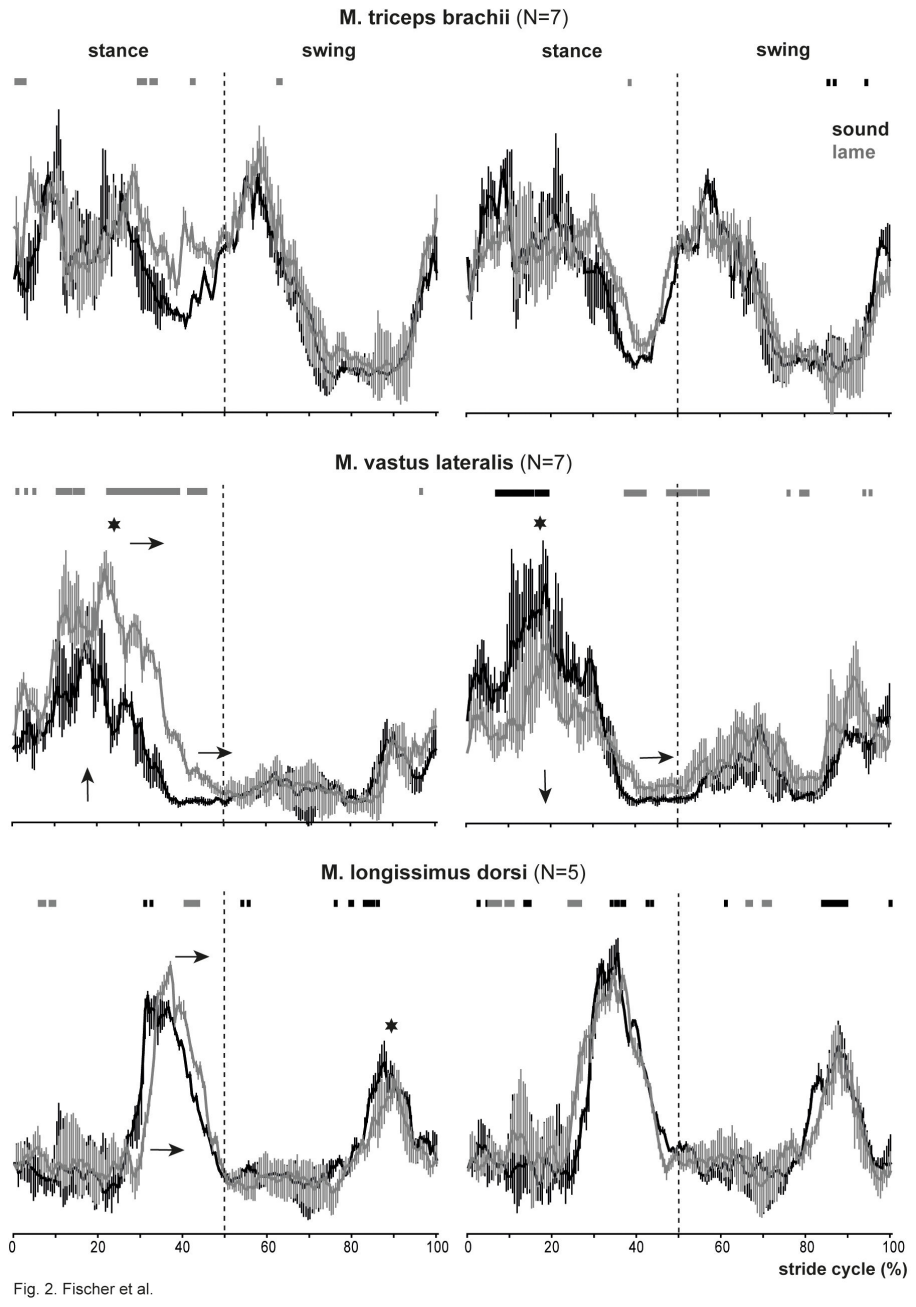
Activity of the m. triceps brachii, m. vastus lateralis, and the m. longissimus dorsi shown as time-normalized SEMGs (median plus upper and lower quartiles for each of the 201 bins) across the dogs and 10 strides per dog for the sound (black) and the lame (grey) conditions. Graphs on the left represent the recordings from the body side contralateral to the lame side; graphs on the right show the activity from the muscles ipsilateral to the lame side. Numbers in parenthesis after the muscle names indicate sample size. Each x-axis shows the stance and swing phase normalized to 50% of the stride cycle in all recordings. The x-axis for the m. triceps brachii refers to the stride cycle of the forelimbs; the x-axes of the m. vastus lateralis and the m. longissimus dorsi refer to the footfall events of the hindlimbs. Each plot has a single y-axis and is scaled to the maximum amplitude observed for that particular recording site; hence SEMG amplitudes can be compared between sound and lame conditions within a given plot. Grey and black blocks above the SEMG traces indicate bin-by-bin differences in amplitude between both conditions, with the color indicating the condition with significantly greater amplitude; no block indicates no differences. The arrows indicate significant differences in the muscle activity between sound and lame conditions: Horizontal arrows point to changes in timing of the on- or offset (bottom of the graph) and the maximum activity (top of the graph). Vertical arrows indicate significant changes in SEMG area. Stars indicate significant differences in peak activity.

The m. triceps brachii showed a biphasic activity. A first activity was observed between late swing and late stance phase. The second burst started shortly before lift off and lasted throughout the first half of the forelimb’s stance phase (Figure 2). Compared with the sound condition, neither the integrated SEMG area nor the timing of the muscle activity was significantly changed in the ipsi- or the contralateral forelimbs (Table 1). 

### *M. vastus lateralis* (N=7)

The m. vastus lateralis was active during most of the stance phase; its activity started during the last quarter of the swing phase and lasted till about 40% of the stride cycle (Figure 2). Compared with the sound condition, muscle activity decreased significantly by 16±15% in the ipsilateral and increased significantly by 66±43% in the contralateral hindlimb after lameness was induced (integrated SEMG area, Table 1). Accordingly, maximum amplitude changed significantly in both hindlimbs. While peak activity occurred significantly later in the contralateral limb, its timing was unchanged in the ipsilateral limb. In both hindlimbs, the duration of the activity increased significantly due to a delayed offset (ipsilateral by 13±6%, contralateral by 29±23% of the respective stride cycle). The bin-wise comparison shows that the increase in activity occurred mainly during the second half of the stance phase in the contralateral limb, while the change in activity in the ipsilateral muscle occurred during the first half of stance (Figure 2). The second and smaller activity associated with the swing phase appeared to increase ipsilaterally (Figure 2), but this difference could not be tested statistically (see Material and Methods). 

### *M. longissimus dorsi (N=5)*

The m. longissimus dorsi was active during the second half of the stance phase and during the second half of the swing phase (Figure 2). Thus, its pronounced biphasic activity ended around lift off and touch down, respectively. Compared to the sound condition, the first and greater burst associated with the stance phase started later and reached its maximum later in the contralateral m. longissimus dorsi (Table 1). Furthermore, peak activity of the second burst was significantly smaller in the contralateral side. All other EMG parameters did not significantly change when hindlimb lameness was induced.

## Discussion

### M. triceps brachii

The m. triceps brachii showed a biphasic activity with a first burst of activity starting shortly before touch down and lasting throughout most of the stance phase and a second activity starting shortly before lift off and ending about halfway through swing phase. Comparisons with previous results from trotting dogs show that the first activity observed in this study agrees well with intramuscular recordings [30-32]. When dogs trot, both the long and the lateral head become active prior to touch down and remain active till mid-stance or during the first two thirds of the stance phase, respectively [30-32]. Therefore, the first activity observed in the current study likely represents a compound signal from the activity of at least the long and the lateral head of the m. triceps brachii. Whether also activity of the accessory head, situated deep and adjacent to the lateral head [33], plays a role is unclear as no recordings from this head exist. Because it is eccentric, the muscle’s activity around touch down has been suggested to control the passive flexion of the elbow joint induced by gravitational forces and allow for elastic energy storage; the subsequent concentric activity extends the elbow joint and thereby provides propulsion during the stance phase [32]. 

In contrast to previous intramuscular recordings in dogs [30,32] and other mammals (e.g., cat [34], horse [35], goat [36]), a second burst associated with the early swing phase was observed in the current study. A function of the biarticular, long head of the m. triceps brachii in shoulder flexion has been suggested based on its topography [33] and shortening pattern [32]. This together with the fact that the timing of the second burst coincides with the flexion of the shoulder joint after lift off (e.g., [32,37,38]) suggests that this activity may be associated with shoulder flexion during the first half of swing. The long head was inactive during early swing phase in previous intramuscular EMG recordings, which may be due to differences in electrode placement among the studies. EMG is a compound signal of the summed action potentials of the muscles fibers located close to the recording site. Therefore, the recorded signal depends on the number and kind of motor units near the electrode, and differences in electrode location may result in differences in the recorded signal (e.g., within the muscle or in vs. on its surface [39]). Furthermore, this second burst could be the result of cross-talk. SEMG does not record the signal directly from the muscle, as does intramuscular EMG; thus, the electrodes detect potentially more than a one muscle signal. Both, the activity [30-32] and the anatomical position [33] of the m. brachialis relative to the electrodes are consistent with the second activity recorded herein. Additionally, skin movements relative to the underlying muscles may lead to slightly different electrode locations during the course of a stride, thus detecting activity from neighboring muscles depending on the inertia of the skin. Dogs, in particular, have relatively loose skin and therefore skin movements may facilitate cross-talk in this species. After lift off, the inertia of the skin places the electrodes slightly more cranially (pers. obs., SF) so that activity, for example, from the m. brachialis may be recorded.

After lameness was induced, neither the intensity nor the timing of the activity of the m. triceps brachii was significantly different. Consistent with this, we observed only minor changes in the vertical force and the temporal gait parameters in the companion study [9]. In agreement with our results in dogs, no kinematic changes occurred in the forelimbs in trotting horses with a transient hindlimb lameness [40] and the recruitment of a forearm muscle (i.e., the m. extensor digitorum longus) was also not significantly different in hindlimb lame horses [41]. 

### M. vastus lateralis

The main activity of the m. vastus lateralis muscle, observed from the last 20% of the swing to ca. 40% of the stance phase, compares very well with previous intramuscular recordings in trotting dogs [30-32] and other mammals (e.g., cat [42,43]; horse [44-46]). This corroborates the previously suggested landmarks for the electrode positioning for this muscle [18,29]. However, compared with intramuscular recordings, our SEMG recordings showed an additional, low-level activity between lift off and about 30% of the swing phase. Because this activity was not observed using intramuscular EMG, it possibly is, as explained above, either the result of differences in the specific electrode location and/or cross-talk. For example, after lift off, the inertia of the skin may place the electrodes slightly more caudally so that activity from the m. biceps femoris is potentially recorded. Furthermore, the m. vastus lateralis is part of the quadriceps muscle group of which both the m. vastus intermedius as well as the m. rectus femoris muscles are in close proximity to the m. vastus lateralis [33]. Activity of all three –the m. biceps femoris caudal, the m. vastus intermedius and the m. rectus femoris- coincide with the burst observed during early swing in the current study [19,22,31,47]. 

After lameness was induced, activity of the ipsilateral m. vastus lateralis was significantly decreased. Consistent with that, the affected limb bears a smaller proportion of the body weight in hindlimb lame dogs [4-9] and several studies reported a substantial loss in muscle mass in the quadriceps group in chronically lame patients (e.g., due to cranial cruciate ligament deficiency [48-52]). Additionally to the decrease in intensity, the timing of the muscle’s activity was changed. That is, the muscle’s activity ended significantly later during stance. Analysis of the temporal gait parameters showed that the stance duration was significantly increased in the affected hindlimb [9], consistent with an increased period of activity. But, the prolonged stance phase may not solely explain the later offset as we performed a stride phase-normalization. However, without detailed kinematic analyses of hindlimb lame dogs, interpretation is hampered. 

Contrary to our results, Bockstahler and colleagues [18] reported a significantly greater activity in the clinically worse limb compared with the contralateral limb. The authors make an increased necessity to stabilize the stifle during early stance and ovoid pain responsible for the greater activity. Elevated stifle stabilization is not required in the load-bearing lameness model used herein, which may explain the differences in the observations between this and the previous study [18]. Additionally, the dogs enrolled in the previous study [18] were patients and thus the time for habituation may have been limited. Unfamiliarity with the experimental situation (e.g., [53]) as well as ‘protective guarding’ for example in anticipation of pain (e.g., [54-56]) lead to substantial changes in muscle recruitment. Alternatively, not mutually exclusive to the above, recruitment patterns and the changes thereof may vary depending on whether a distal, supporting lameness (this study) or a proximal lameness due to hip osteoarthritis exists [18].

In the contralateral m. vastus lateralis, both the intensity and the duration of the activity of were significantly increased after lameness was induced. Accordingly, this limb bears a greater proportion of the body weight in trotting dogs when lame and its stance duration is significantly increased (e.g., [4-7,9]). As the bin-wise comparison shows, muscle activity was primarily increased during the second half of stance; that is, when the hindlimb exerts propulsive forces and muscles activity is concentric to produce knee extension [32,37,38]. Therefore, the increased activity during the second half of stance is most likely associated with the pronounced production of knee extension to propel the body forward. Accordingly, in dogs with hindlimb lameness, the sound limb produces a greater share of the propulsive forces in order to compensate for the lost function of the other limb [57,58]. 

### M. longissimus dorsi

The mid-lumbar SEMG recordings of this study showed the typical biphasic activity with a greater burst during the ipsilateral stance phase and a smaller one during the ipsilateral swing phase that is well-documented for the m. longissimus dorsi in several mammals (e.g., dog [21,23,24,30,31]; cat [59]; horse [60-64]). Because our results agree well with previous recordings from this muscle, the anatomical landmarks used are well-suited electrode positions. Nevertheless, it should be kept in mind that the activity of the mid-lumbar m. longissimus dorsi and m. multifidus are very similar [21,24]. Therefore, potential cross-talk can not be easily detected and the recorded signal potentially represents the summed activity of these epaxial muscles. 

Because the activity on one body side coincides with the activity on the other side, bilateral activity results. Bilateral activity is required to mobilize and stabilize the trunk in the sagittal plane [23]. Manipulations of the locomotor forces in trotting dogs showed that the bilateral activity stabilizes the trunk against the inertia of the CoM (‘sagittal rebound’, [21]) and the vertical components of the extrinsic hindlimb muscles (e.g., hindlimb retractors, [24]). Assuming that dogs manage hindlimb lameness like horses, the reduced vertical acceleration of the CoM associated with the lame hindlimb’s stance phase and the compensatory increase of CoM motions during the sound hindlimb’s stance [65,66] should result in diagonally opposite but alike changes of the two corresponding bursts. For example, an increased need to stabilize the trunk in the sagittal plane can be expected to be associated with an increased first burst of the ipsilateral and an increased second burst of the contralateral muscle (i.e., the two bursts which coincide on the two sides). Because the corresponding bursts did not show concordant changes after lameness induction, rather all significant changes concerned the unilaterally greater activity, changes in the forces acting in the sagittal plane may have been too small to result in substantial changes in the muscle activity in this study. 

In sound trotting dogs, the unilaterally greater activity acts to stabilize the trunk in the transverse plane against gravitational forces and in the horizontal plane against the horizontal components of the extrinsic hindlimb muscles [24]. Pronounced long-axis rotations of the pelvis and trunk towards the sound side were observed in lame horses and suggested as one means to unload the affected limb [65,67]. To produce these rotations, increased activity of the epaxial as well as the extrinsic limb muscles (i.e., the m. gluteus medius) contralateral to the affected side can be expected [22,24]. Although not significant, the results of this study show an increased first activity of the m. longissimus dorsi contralateral to the lame limb, consistent with pronounced long-axis rotation to towards the sound side. Increased activity of m. gluteus medius is furthermore expected because of the changes in limb trajectory; that is, hindlimb lame animals such as horses skew their limbs medio-laterally in order to move the sound limb more directly under the CoM [40]. In agreement with that, greater activity was observed in this muscle in lame horses during walking [41]. 

Because the unilateral epaxial muscle activity is also associated with the stabilization of the pelvis against the action of the ipsilateral protractor and the contralateral retractor of the hindlimb [24], changes in timing and/or the amplitude of limb motions likely cause changes in the epaxial muscle activity. Unfortunately, no detailed kinematic analyses are available for dogs with distal, weight-bearing hindlimb lameness. In horses, however, the sound hindlimb’s protraction is delayed [40], consistent with the delayed onset and peak activity of the m. longissimus dorsi observed herein. 

### Clinical relevance

Intramuscular EMG has been widely used in basic research to document the activity of various limb and back muscles (e.g., sound trotting dogs [19,21-24,30-32,68,69]), but only very few studies used it to evaluate muscular adaptions to lameness [18,41,70]. To limit the invasiveness during data collection, supracutaneous recordings using surface electrodes are commonly obtained from human patients [71]. In animal patients, SEMG has only recently been used to study muscle function in sound [60,61,72] and lame horses [41] as well as in sound [29,73] and lame dogs [18] (this study). Compared to human subjects, cooperation and tolerance to skin manipulation, but also differences in skin properties (e.g., tightness) may have hindered a more widespread use of SEMG as a diagnostic tool in animals.

The changes in muscle activity observed in the current study were consistent with previously described alterations in kinematic and kinetic parameters as well as in muscle mass in chronically lame patients. Nevertheless, one must keep in mind that the results obtained using this induced lameness model (i.e., distal, short-term, load-bearing lameness) may differ from the changes occurring in patients lame due to diseases. Independent from the cause for lameness, however, changes in muscle function trigger more gradual tissue responses such as tissue remodeling [3]. Because muscular forces primarily determine joint loading, changes in muscle activity potentially also cause skeletal remodeling or joint degeneration [70]. In addition, joint stabilization *via* co-contraction may result in changed joint forces despite similar limb trajectories and/or comparable unloading of the limb [74]. Hence, analyzing muscle activity provides diagnostic information into gait changes in addition to the well-established parameters and has prognostic value because it allows insight into the short- and long-term effects of altered functional demands on the musculoskeletal system. Furthermore, a better understanding of the adaptations in muscle activity to lameness has the potential to improve treatment options and rehabilitative exercises for chronic patients (e.g., by developing targeted muscle training). At present, kinesiological EMG is on the fringe of veterinary medicine, but its proven benefit as a tool in basic and applied research, physiotherapy, rehabilitation, and sports training in humans should encourage its broader establishment and application in veterinary sciences. 
